# Evolution of illness severity in hospital admissions due to COVID-19, Québec, Canada, January to April 2022

**DOI:** 10.14745/ccdr.v50i12a08

**Published:** 2024-01-01

**Authors:** Ernest Lo, Élise Fortin, Rodica Gilca, Pierre-Luc Trépanier, Hany Geagea, Zhou Zhou

**Affiliations:** 1 Institut national de santé publique du Québec, Québec, QC; 2 Department of Epidemiology, Biostatistics and Occupational Health, McGill University, Montréal, QC; 3 Département de microbiologie, Infectiologie et immunologie, Faculté de médecine, Université de Montréal, Québec, QC; 4 Département de médecine sociale et préventive, Faculté de médecine, Université Laval, Québec, QC; 5 Centre de recherche du CHU de Québec, Université Laval, Québec, QC

**Keywords:** COVID-19, hospitalizations, severity, surveillance

## Abstract

**Background:**

The coronavirus disease 2019 (COVID-19) severity is influenced by multiple factors, such as age, underlying medical conditions, individual immunity, infecting variant, and clinical practice. The highly transmissible Omicron variants resulted in decreased COVID-19 screening capacity, which limited disease severity surveillance.

**Objective:**

To report on the temporal evolution of disease severity among patients admitted to Québec hospitals due to COVID-19 between January 2, 2022, and April 23, 2022, which corresponded to the peak period of hospitalizations due to Omicron.

**Methods:**

Retrospective population-based cohort study of all hospital admissions due to COVID-19 in Québec, between January 2, 2022, and April 23, 2022. Study period was divided into four-week periods, corresponding roughly to January, February, March and April. Regression using Cox and Poisson generalized estimating equations (GEEs) was used to quantify temporal variations in length of stay and risk of complications (intensive care admission or in-hospital death) through time, using the Omicron peak (January 2022) as reference. Measures were adjusted for age, sex, vaccination status, presence of chronic diseases, and clustering by hospital.

**Results:**

During the study period, 9,178 of all 18,272 (50.2%) patients hospitalized with a COVID-19 diagnosis were admitted due to COVID-19. Of these, 1,026 (11.2%) were admitted to intensive care and 1,523 (16.6%) died. Compared to January, the risk of intensive care admission was 25% and 31% lower in March and April respectively, while in-hospital fatality continuously decreased by 45% lower in April. The average length of stay was temporarily lower in March (9%).

**Conclusion:**

Severity of admissions due to COVID-19 decreased in the first months of 2022, when predominant circulating variants were considered to be of similar severity. Monitoring hospital admissions due to COVID-19 can contribute to disease severity surveillance.

## Introduction

When a new severe acute respiratory syndrome coronavirus 2 (SARS-CoV-2) variant or sublineage appears, efforts are made to rapidly characterize its transmissibility and severity compared to previous variants. The Omicron BA.1 variant was first detected in Québec on December 8, 2021, during the Delta wave of the pandemic, and became predominant by December 12, 2021. Subsequently, the Omicron BA.2 variant appeared on January 1, 2022, and was predominant by March 27, 2022. A peak in hospital admissions due to SARS-CoV-2 (the coronavirus that causes COVID-19) was recorded on January 18, 2022 ((1)). Overall, the Omicron variant had higher transmissibility but lower severity compared to the Delta variant ((2–6)), while the Omicron BA.1 and BA.2 sublineages had comparable severity ((5,7–9)). Such information is essential for public health teams to help them anticipate the evolution of the epidemic, including the new variant’s impact on healthcare resources. In Canada, where the number of hospital beds per inhabitant is low and the workforce has been affected by the COVID-19 pandemic, information on severity will help to determine whether or not public health measures should be applied or maintained ((10,11)).

Severity of COVID-19 cases depends on factors beyond the characteristics of the virus. In times of high incidence, more hospitalizations will occur and the threshold for hospital admission/discharge might change, regardless of virulence (12). Natural, vaccine-induced, and hybrid immunity have increased in the population since the beginning of the COVID-19 pandemic but will vary according to time since infection or vaccination (13–15). Clinical care has also evolved with increasing knowledge and experience in treatment, as well as with the arrival of antiviral treatments ((16,17)). Finally, with the explosion of cases following the emergence of Omicron variants and the availability of rapid tests, accurate estimates of the total number of cases and, consequently, the proportion of severe disease in the general population, were no longer possible. In contrast, all patients admitted to hospital in the province of Québec receive a PCR test for COVID-19, a practice that was consistent throughout the pandemic ((18)). Propensity of hospitalization given a certain level of severity in Québec also was not impacted by the adoption of rapid tests. Thus, tracking the evolution of the severity of cases among those admitted to hospital due to COVID-19 represents a potentially interesting alternative for disease severity monitoring.

We aimed to describe the severity of hospital admissions due to COVID-19 in Québec between January 2022 and April 2022, which corresponded to the Omicron BA.1 and BA.2 waves. We measured length of stay, risk of intensive care admission, and risk of in-hospital death, and quantified temporal variations of these measures.

## Methods

### Study design and population

A retrospective population-based cohort was built using linked data to study all Québec hospitalizations for which COVID-19 led to hospital admission between January 2, 2022, and April 23, 2022 (Centers for Disease Control and Prevention, weeks 1–16). Patients were followed from admission until discharge, death, or final date of data extraction (May 25, 2022).

### Data sources and variables

**Figure 1:**
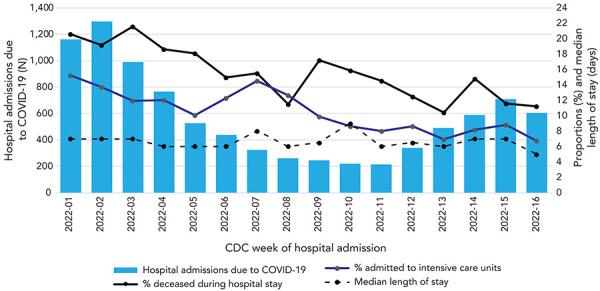
Time trends in hospital admissions due to COVID-19, median length of stay and frequency of complications, January 2, 2022, and April 23, 2022, Québec Abbreviations: CDC, Centers for Disease Control and Prevention; COVID-19, coronavirus disease 2019

The COVID-19 hospitalizations were identified using the provincial hospital admissions database, which is a real-time version of the provincial hospital discharge database (MED-ECHO) routinely available before the pandemic. For this real-time database, hospital medical archivists reported any presence of COVID-19 during a hospital stay, regardless of other health conditions. Since December 30, 2021, archivists also provided admission diagnosis for all patients with a COVID-19 diagnosis during their hospital stay. Admission and hospital stay diagnoses are recorded according to the International Classification of Diseases 10^th^ Revision (ICD-10). Among all patients with a COVID-19 diagnosis during their hospital stay, those with an admission code related to COVID-19 were identified as admissions due to COVID-19. The list of COVID-19-related diagnostic codes used in provincial surveillance is provided in **Appendix**, **Table A1**. In addition to admission diagnosis, admission and discharge dates, age, sex, intensive care admissions, and death while hospitalized are also recorded in this database. The study period was divided into four four-week periods that corresponded to the peak (January) and the tail (February) of the BA.1 wave, the transition towards BA.2 (March), and the beginning and peak of the BA.2 wave (April) (**Figure 1**). Using a unique identifier, the hospitalization database was linked to:

• The Québec Integrated Chronic Diseases Surveillance System to identify patients with at least one of 31 comorbidities ((19))

• The provincial laboratory database to identify patients who had a positive SARS-CoV-2 test more than 90 days before the current admission (interpreted as a reinfection)

• The provincial immunization registry for information on COVID-19 vaccination status (individuals with at least two doses were considered adequately vaccinated)

### Analyses

The proportion of admissions due to COVID-19 with an intensive care admission, as well as the proportion of patient deaths, were computed for each time period. The 25^th^, 50^th^ and 75^th^ percentiles of length of stay were additionally computed, once again for each time period. Although length of stay was censored after 28 days, the estimated percentiles were always less than 28 days and so were unaffected. These proportions and duration were also stratified by age group (0–45, 46–55, 56–65, 66–75 and over 75 years old), sex, vaccination status, history of COVID-19 infection, and presence or absence of comorbidities, respectively.

Regression analyses were used to quantify the association between both length of stay and risk of complications (intensive care admission or death) vs. time period, using the Omicron peak period (January 2022) as reference. Cox proportional hazards regression was used to analyze length of stay, using random effects to model the possible clustering effect of hospitals. General estimating equations using a Poisson distribution and exchangeable correlation matrix were used to analyze risk of intensive care admission and death, accounting for the possible clustering effect of hospitals. For the above regressions, unadjusted and fully adjusted associations with time period are presented. Fully adjusted models used age group, sex, vaccination status, and chronic disease as covariates; no adjustment was made for history of COVID-19 infection because patients hospitalized for a reinfection were too rare (less than 3% of patients hospitalized for COVID-19). To isolate changes in disease severity from patient immunity to the disease, additional models were produced only for patients who had no known history of COVID-19 infection and who were not vaccinated. In subgroup analyses, separate models were also produced for each age group. Finally, after learning that three hospitals had largely underestimated intensive care admissions in early 2022, the fully adjusted regression was done, excluding these three hospitals, in a *post hoc* sensitivity analysis. All analyses were done using R 4.0.2; mixed effects Cox regression was done using the coxme package ((20)), while Poisson GEE was done using the geepack package ((21)).

## Results

Between January 2, 2022, and April 23, 2022, 9,178 (50.2%) of all 18,272 patients who were hospitalized with a COVID-19 diagnosis were admitted due to COVID-19. Of these, 1,026 (11.2%) were admitted to an intensive care unit and 1,523 (16.6%) died while hospitalized (these outcomes were not mutually exclusive). Slightly over half of patients admitted due to COVID-19 were male (52.8%), and a majority of patients were over 65 years old (72.2%), adequately vaccinated (72.1%), experiencing their first known SARS-CoV-2 infection (98.1%) and had at least one comorbidity (83.7%). These statistics are described per four-week period in **Table 1** (see **Table A2** and **Table A3** for a description of intensive care unit admissions and deaths per four-week period). Patient characteristics were relatively stable over time, except for a higher proportion of older patients and a lower proportion of inadequately vaccinated patients in March and April. In any given group, patients still hospitalized after 28 days represented less than 10% of inpatients.

**Table 1:** Description of hospital admissions due to COVID-19, by four-week periods, January 2, 2022, and April 23, 2022, Québec

Variable	January 2 to January 29, 2022		January 30 to February 26, 2022		February 27 to March 26, 2022		March 27 to April 23, 2022	
	n	%	n	%	n	%	n	%
**Global**	**4,216**	**100.0**	**1,550**	**100.0**	**1,015**	**100.0**	**2,397**	**100.0**
Admitted to ICU	565	13.4	187	12.1	89	8.8	185	7.7
In-hospital death	844	20.0	241	15.5	150	14.8	288	12.0
**Age group (years)**								
0–45	469	11.1	247	15.9	121	11.9	248	10.3
44–55	254	6.0	89	5.7	39	3.8	68	2.8
56–65	544	12.9	171	11.0	103	10.1	200	8.3
66–75	911	21.6	316	20.4	186	18.3	446	18.6
Over 75	2,038	48.3	727	46.9	566	55.8	1,435	59.9
**Sex**								
Male	2,252	53.4	809	52.2	538	53.0	1,243	51.9
Female	1,964	46.6	741	47.8	477	47.0	1,154	48.1
**Vaccination**								
Adequate	2,864	67.9	1,027	66.3	785	77.3	1,940	80.9
Inadequate	1,348	32.0	519	33.5	229	22.6	453	18.9
Missing information (or Unknown)	4	0.1	4	0.3	1	0.1	4	0.2
**Prior infection according to laboratory tests**								
No	4,151	98.5	1,520	98.1	993	97.8	2,338	97.5
Yes	65	1.5	30	1.9	22	2.2	59	2.5
**Comorbidities**								
None	521	12.4	219	14.1	122	12.0	236	9.8
At least one	3,526	83.6	1,247	80.5	846	83.3	2,066	86.2
Missing	169	4.0	84	5.4	47	4.6	95	4.0

Abbreviations: COVID-19, coronavirus disease 2019; ICU, intensive care unit

Globally, patients were more frequently admitted to intensive care units in January and February, while length of stay remained relatively stable over time (Figure 1). Patients also died more frequently during the January peak of hospital admissions, with a gradual decrease throughout the following weeks (Figure 1). These time trends were also observed in regression analyses, after adjusting for age, sex, vaccination status, and presence of at least one comorbidity (**Table 2**). The proportions of patients admitted to intensive care were 25% and 31% lower in March and April (peak of BA.2; Table 1), respectively, as compared with the Omicron peak (January); this trend towards a risk reduction in time was observed in all age groups except for those 0–45 years old (**Table 3**). Results were similar when excluding the three hospitals that underestimated intensive care admissions (adjusted risk ratios of 0.92, 0.76 and 0.70 for February, March and April, respectively). The proportion of in-hospital deaths decreased continuously and was 45% lower in April, compared to January (Table 2); this trend was driven by patients over 75 years old, as 78% of deaths occurred in this age group (Table A3). In non-vaccinated patients admitted for a first COVID-19 episode, adjusted time trends in risk of intensive care admission and in-hospital death were very similar to those observed in the entire cohort (Table 2). Finally, the probability of remaining in hospital after any given number of days was 9% lower in March (transition towards BA.2) compared to January (BA.1 peak), but this was a temporary decrease (Table 2). No statistically significant change in length of stay was observed for hospitalizations of non-vaccinated patients. Cox regressions stratified by age group had extremely high statistical variability, indicating both increasing or decreasing lengths of stay (Table 3).

**Table 2:** Evolution of length of stay^a^, proportions of patients admitted to ICU^b^ and in-hospital deaths^b^ among hospital admissions due to COVID-19, January 2, 2022, and April 23, 2022, Québec

Population type by time period	Length of stay		Intensive care admissions		In-hospital deaths	
	Unadjusted hazard ratio (95% CI)	Fully adjusted^a^ hazard ratio (95% CI)	Unadjusted proportion ratio (95% CI)	Fully adjusted^c^ proportion ratio (95% CI)	Unadjusted proportion ratio (95% CI)	Fully adjusted^c^ proportion ratio (95% CI)
**Global**						
January 2 to January 29, 2022	Reference	Reference	Reference	Reference	Reference	Reference
January 30 to February 26, 2022	1.01 (0.95–1.07)	1.01 (0.95–1.07)	0.90 (0.77–1.05)	0.91 (0.78–1.07)	0.78 (0.69–0.89)	0.81 (0.71–0.92)
February 27 to March 26, 2022	0.88 (0.82–0.94)	0.91 (0.84–0.97)	0.66 (0.53–0.82)	0.75 (0.61–0.93)	0.73 (0.63–0.86)	0.70 (0.60–0.82)
March 27 to April 23, 2022	0.99 (0.94–1.04)	1.03 (0.97–1.08)	0.57 (0.48–0.67)	0.69 (0.58–0.80)	0.60 (0.53–0.68)	0.55 (0.48–0.62)
**Unvaccinated with no previous COVID-19 infection**						
January 2 to January 29, 2022	Reference	Reference	Reference	Reference	Reference	Reference
January 30 to February 26, 2022	1.15 (1.01–1.31)	1.08 (0.95–1.22)	1.04 (0.82–1.32)	1.06 (0.83–1.34)	0.92 (0.69–1.21)	0.88 (0.68–1.15)
February 27 to March 26, 2022	1.03 (0.85–1.25)	0.96 (0.80–1.17)	0.74 (0.48–1.12)	0.73 (0.48–1.12)	0.66 (0.40–1.07)	0.74 (0.47–1.17)
March 27 to April 23, 2022	0.98 (0.85–1.13)	0.88 (0.76–1.01)	0.69 (0.51–0.95)	0.71 (0.52–0.97)	0.65 (0.46–0.92)	0.57 (0.41–0.81)

Abbreviations: CI, confidence interval; COVID-19, coronavirus disease 2019

^a^ Hazard ratios

^b^ Proportion ratios

^c^ Adjusted for age group, sex, vaccination status and presence or absence of comorbidities

**Table 3:** Evolution of length of stay^a^, proportions of patients admitted to ICU^b^ and in-hospital deaths^b^ among hospital admissions due to COVID-19, by age group, January 2 and April 23, 2022, Québec

Age by time period	Length of stay	Intensive care admissions	In-hospital deaths
	Fully adjusted^c^ hazard ratio (95% CI)	Fully adjusted^c^ proportion ratio (95% CI)	Fully adjusted^c^ proportion ratio (95% CI)
**0–45 years**			
January 2 to January 29, 2022	Reference	Reference	Reference
January 30 to February 26, 2022	1.3 (0.93–1.83)	0.65 (0.40–1.05)	1.17 (0.29–4.77)
February 27, 2022, to March 26, 2022	1.12 (0.72–1.73)	0.50 (0.25–1.02)	0.81 (0.09–7.06)
March 27, 2022, to April 23, 2022	1.2 (0.89–1.87)	0.77 (0.48–1.24)	2.20 (0.72–6.68)
**46–55 years**			
January 2 to January 29, 2022	Reference	Reference	Reference
January 30 to February 26, 2022	1.08 (0.85–1.37)	1.19 (0.73–1.93)	1.47 (0.50–4.31)
February 27 to March 26, 2022	1.62 (1.19–2.22)	0.93 (0.45–1.95)	1.44 (0.35–5.96)
March 27 to April 23, 2022	1.07 (0.83–1.38)	0.26 (0.08–0.83)	0
**56–65 years**			
January 2 to January 29, 2022	Reference	Reference	Reference
January 30 to February 26, 2022	0.97 (0.84–1.12)	0.81 (0.58–1.14)	0.88 (0.52–1.48)
February 27 to March 26, 2022	0.86 (0.71–1.04)	0.78 (0.50–1.22)	0.73 (0.36–1.47)
March 27 to April 23, 2022	1.13 (0.98–1.31)	0.61 (0.42–0.89)	0.63 (0.36–1.11)
**66–75 years**			
January 2 to January 29, 2022	Reference	Reference	Reference
January 30 to February 26, 2022	1.03 (0.9–1.17)	0.95 (0.73–1.24)	0.88 (0.66–1.18)
February 27 to March 26, 2022	0.86 (0.73–1.01)	0.84 (0.58–1.22)	0.85 (0.59–1.24)
March 27 to April 23, 2022	1.22 (1.08–1.38)	0.74 (0.56–0.97)	0.54 (0.39–0.75)
**Over 75 years**			
January 2 to January 29, 2022	Reference	Reference	Reference
January 30 to February 26, 2022	0.95 (0.87–1.04)	1.04 (0.77–1.40)	0.77 (0.67–0.90)
February 27 to March 26, 2022	0.85 (0.77–0.94)	0.76 (0.51–1.13)	0.67 (0.56–0.80)
March 27 to April 23, 2022	0.92 (0.86–0.99)	0.73 (0.55–0.96)	0.53 (0.46–0.61)

Abbreviations: CI, confidence interval; COVID-19, coronavirus disease 2019

^a^ Hazard ratios

^b^ Proportion ratios

^c^ Adjusted for sex, vaccination status and presence or absence of comorbidities

## Discussion

This study showed a decreasing trend in the risks of intensive care admission and in-hospital death among patients admitted to hospital due to COVID-19 in Québec throughout the first 16 weeks of 2022. No clear trend emerged with respect to temporal variations in the length of hospital stay. Conclusions were similar in sensitivity analyses focusing on unvaccinated patients with no previous documented COVID-19 infection.

Many factors may have contributed to decreasing severity. Patient age, sex and comorbidities have been identified as risk factors for severe outcomes in the early stages of the pandemic ((12,22–24)), but analyses for these factors were adjusted and/or stratified, as well as controlled for vaccination status. Residual confounding may nevertheless remain. Xia *et al.* reported a positive association between in-hospital mortality (in all COVID-19-positive inpatients) and the proportion of available beds occupied by COVID-19-positive patients in Québec, during the first three waves of the pandemic ((12)). The arrival of the Omicron variant led to the highest number of patients hospitalized with a COVID-19 diagnosis since the beginning of the pandemic ((1)). This patient load may also have contributed to the trends observed in our study. However, the last four-week period included the peak of the BA.2 wave, and severity kept decreasing even though an increase would have been expected given the higher number of admissions. It is possible that this phenomenon may still have occurred but was not strong enough to reverse the overall trend. Clinical practices also keep evolving, with antiviral treatments becoming available at the beginning of the study period and with increasing accessibility over time ((17,25)). However, without access to patient load, healthcare worker absenteeism, or antiviral use data, these variables could not be accounted for. Finally, social determinants of health, which represent a well-known driver of inequalities in COVID-19 susceptibility and outcomes, were not accounted for in these analyses ((26)). However, the effect of social determinants is likely controlled for in the regression analyses, at least in part, through other covariates, such as comorbidities and vaccination status.

Variant composition also evolved during the study period and could have contributed to observed severity trends. Delta-infected patients were still being admitted to hospital in early January, which could explain a higher in-hospital mortality during the first four-week period, but not the decrease in severity observed for the last two periods (27). Estimates of the severity of the BA.1 and BA.2 sublineages have suggested a possible lower severity of BA.2 ((5,7,8)), though differences measured within each study were not statistically significant. Whole genome sequencing data were unavailable for hospitalized patients; therefore, an association between observed severity trends and variant composition could not be confirmed. Other possible factors are that patients from the more recent periods had shorter follow-up and thus less time to experience outcomes (discharge, intensive care unit admission or death), as not all inpatients had been discharged by the end of the study period. However, all patients were followed for at least 28 days, which should be sufficient to capture the majority of outcomes. The practice of PCR testing of all the patients admitted to hospital in Québec ((18)) also rules out changes in testing practices as a factor in severity trends.

In the time preceding the study, PCR testing was done in the general population; nevertheless, not all cases, especially if mild or asymptomatic, were necessarily detected. Therefore, reinfection or the presence of previous COVID-19 infection could have gone undetected in some patients. However, this would only affect severity trends if the proportion of undetected reinfections varied over time. Overall, COVID-19 testing quality and coverage in Québec were high before December 2021 and the advent of Omicron. It is possible, however, that the proportion of hospital patients with unmeasured previous COVID infection acquired during or after December 2021 could have contributed to the observed decreasing severity for the month of April, since a previous infection is defined as one that occurs at least three months before the testing date. Finally, it is possible that the “adequate vaccination” criterion used in the regression analyses does not account for the effect of waning vaccine efficacy, which could result in misclassification of patients that were thought to be protected due to vaccine immunity. However, this effect is likely minimal, given that the majority (84%) of adequately vaccinated patients in this study received their last dose within seven months of hospital admission. This seven-month threshold is based on vaccine effectiveness studies ((28)). Sensitivity analyses (not shown), where patients receiving their last dose more than seven months after admission to hospital were classified as inadequately vaccinated, showed negligible difference in estimated severity trends.

When Omicron hit the province of Québec in December 2021, screening clinics and laboratories were quickly overloaded. January 2022 marked the end of two years of universal screening. At this time, a new screening strategy was adopted that targeted only certain subpopulations, mostly consisting of the elderly, especially in long-term care facilities, healthcare workers, and patients admitted to hospital ((29)). Surveillance of disease severity by following up on COVID-19 cases until hospital admission or death would therefore have been biased given the reasons behind the selection of these groups (e.g., increased vulnerability, higher exposure to disease and to vaccines, and the healthy worker effect). Monitoring severity among inpatients represented an alternative because all inpatients were still tested. Our previous work on disease severity comparing Omicron and Delta variants among inpatients suggested a lower severity of Omicron hospitalizations, concordant with other studies comparing these two variants with different methodologies ((3–6,30)). Wolter *et al.* reached convergent conclusions regarding the relative severity of BA.1 and BA.2 sublineages by measuring and comparing the difference in both risk of hospital admission among cases and risk of severe outcomes among inpatients ((8)).

The restriction of analyses only to patients admitted due to COVID-19 is an important strength of this study, as about half of all COVID-19-positive inpatients were admitted for other illnesses that can differ in severity from COVID-19. As well, healthcare-associated cases of COVID-19, which are more frequent in periods of high viral circulation, have been related to more severe outcomes ((31,32)). Unfortunately, admission diagnosis was only available from December 30, 2021, which prevented a comparison of Omicron waves with earlier waves. Before January 2022, all COVID-19-positive patients were analyzed, with the finding that median length of stay, proportion admitted to intensive care, and proportion of in-hospital deaths all varied in a similar manner over time, suggesting that length of stay could be used to inform disease severity (30). This correspondence was not observed in the present analysis, however. Length of stay may be influenced by patient load during peaks and its utility for surveillance of severity is therefore unclear. Also, the results of this study do not provide information on the effect of interventions that aim to prevent hospitalizations. For instance, compared to the general population, hospitalized cases over-represent individuals where vaccines and antivirals have not been successful. Finally, as was previously pointed out by Twohig *et al.*, this surveillance informs the evolution of severity with a delay, as admissions follow case onset by a few days and as a majority of patients have to be discharged before intensive care admissions, in-hospital deaths, and length of stay can be assessed ((22)).

## Conclusion

Throughout the first months of 2022, the risks of in-hospital death or intensive care admission decreased in individuals admitted due to COVID-19. Many factors, including changing immunity, reinfection prevalence, antiviral usage, and patient load may have contributed to this trend, which occurred during a time when virulence of predominant circulating variants were not excessively different. Hospital admissions due to COVID-19 represent an opportunity for monitoring trends in disease severity.

## Appendix

**Table A1:** List of COVID-19-related diagnostic codes (ICD-10) used in provincial surveillance, Quebec

Code	Description
A090	Gastroentérite et colite autre et non précisée d'origine infectieuse
A099	Gastroentérite et colite d'origine non précisée
A418	Autres sepsies précisées
A419	Sepsie, sans précision
A498	Autres infections bactériennes, siège non précisé
A499	Infection bactérienne, sans précision
B348	Autres infections virales, siège non précisé
B349	Infection virale, sans précision
E860	Déshydratation
G430	Migraine sans aura [migraine commune]
G431	Migraine avec aura [migraine classique]
G432	État de mal migraineux
G433	Migraine compliquée
G438	Autres migraines
G439	Migraine, sans précision
G441	Céphalée vasculaire, non classée ailleurs
G442	Céphalée dite de tension
G444	Céphalée médicamenteuse, non classée ailleurs
G448	Autres syndromes précisés d’algies céphaliques
G933	Syndrome de fatigue post-virale
I260	Embolie pulmonaire, avec mention de coeur pulmonaire aigu
I269	Embolie pulmonaire, sans mention de coeur pulmonaire aigu
J00	Rhinopharyngite aiguë [rhume banal]
J010	Sinusite maxillaire aiguë
J011	Sinusite frontale aiguë
J012	Sinusite ethmoïdale aiguë
J013	Sinusite sphénoïdale aiguë
J014	Pansinusite aiguë
J018	Autres sinusites aiguës
J019	Sinusite aiguë, sans précision
J020	Pharyngite à streptocoques
J028	Pharyngite aiguë due à d’autres micro-organismes précisés
J029	Pharyngite aiguë, sans précision
J040	Laryngite aiguë
J041	Trachéite aiguë
J042	Laryngo-trachéite aiguë
J050	Laryngite obstructive aiguë [croup]
J051	Épiglottite aiguë
J060	Laryngo-pharyngite aiguë
J068	Autres infections aiguës des voies respiratoires supérieures, à localisations multiples
J069	Infection des voies respiratoires supérieures, sans précision
J09	Grippe, due à un virus grippal zoonotique ou pandémique identifié
J110	Grippe avec pneumonie, virus non identifié
J111	Grippe avec d'autres manifestations respiratoires, virus non identifié
J118	Grippe avec d’autres manifestations, virus non identifié
J120	Pneumonie adénovirale
J121	Pneumonie due au virus respiratoire syncytial [VRS]
J122	Pneumonie due aux virus paragrippaux
J123	Pneumonie due au métapneumovirus humain
J128	Autre pneumonie virale
J129	Pneumonie virale, sans précision
J13	Pneumonie due à *Streptococcus pneumoniae*
J14	Pneumonie due à *Haemophilus influenzae*
J150	Pneumonie due à *Klebsiella pneumoniae*
J151	Pneumonie due à Pseudomonas
J152	Pneumonie due à des staphylocoques
J153	Pneumonie due à des streptocoques, groupe B
J154	Pneumonie due à d’autres streptocoques
J155	Pneumonie due à *Escherichia coli*
J156	Pneumonie due à d’autres bactéries à Gram négatif
J157	Pneumonie due à *Mycoplasma pneumoniae*
J158	Autres pneumonies bactériennes
J159	Pneumonie bactérienne, sans précision
J160	Pneumonie due à Chlamydia
J168	Pneumonie due à d’autres micro-organismes infectieux
J170	Pneumonie au cours de maladies bactériennes classées ailleurs
J171	Pneumonie au cours de maladies virales classées ailleurs
J172	Pneumonie au cours de mycoses
J173	Pneumonie au cours de maladies parasitaires
J178	Pneumonie au cours d’autres maladies classées ailleurs
J180	Bronchopneumonie, sans précision
J181	Pneumonie lobaire, sans précision
J182	Pneumonie hypostatique, sans précision
J188	Autre pneumonie, micro-organisme non précisé
J189	Pneumonie, sans précision
J200	Bronchite aiguë due à *Mycoplasma pneumoniae*
J201	Bronchite aiguë due à *Haemophilus influenzae*
J202	Bronchite aiguë due à des streptocoques
J203	Bronchite aiguë due au virus Coxsackie
J204	Bronchite aiguë due aux virus paragrippaux
J205	Bronchite aiguë due au virus respiratoire syncytial [VRS]
J206	Bronchite aiguë due à des rhinovirus
J207	Bronchite aiguë due à des virus ECHO
J2080	Bronchite aiguë due au métapneumovirus humain
J2088	Bronchite aiguë due à d’autres micro-organismes précisés
J209	Bronchite aiguë, sans précision
J210	Bronchiolite aiguë due au virus respiratoire syncytial [VRS]
J211	Bronchiolite aiguë due au métapneumovirus humain
J218	Bronchiolite aiguë due à d’autres micro-organismes précisés
J219	Bronchiolite aiguë, sans précision
J22	Infection aiguë des voies respiratoires inférieures, sans précision
J398	Autres maladies des voies respiratoires supérieures précisées
J399	Maladie des voies respiratoires supérieures, sans précision
J40	Bronchite, non précisée comme aiguë ou chronique
J440	Maladie pulmonaire obstructive chronique avec infection aiguë des voies respiratoires inférieures
J441	Maladie pulmonaire obstructive chronique avec exacerbation aiguë, sans précision
J448	Autres maladies pulmonaires obstructives chroniques précisées
J449	Maladie pulmonaire obstructive chronique, sans précision
J80	Syndrome de détresse respiratoire de l'adulte
J90	Épanchement pleural, non classé ailleurs
J91	Épanchement pleural au cours de maladies classées ailleurs
J960	Insuffisance respiratoire aiguë
J9600	Insuffisance respiratoire aiguë Type I [hypoxique]
J9601	Insuffisance respiratoire aiguë Type II [hypercapnique]
J9609	Insuffisance respiratoire aiguë, type non précisé
J961	Insuffisance respiratoire chronique
J9610	Insuffisance respiratoire chronique Type I [hypoxique]
J9611	Insuffisance respiratoire chronique Type II [hypercapnique]
J9619	Insuffisance respiratoire chronique, type non précisé
J969	Insuffisance respiratoire, sans précision
J9690	Insuffisance respiratoire, sans précision, type I [hypoxique]
J9691	Insuffisance respiratoire, sans précision, Type II [hypercapnique]
J9699	Insuffisance respiratoire, sans précision, type non précisé
J980	Affections des bronches, non classées ailleurs
J984	Autres affections pulmonaires
J988	Autres troubles respiratoires précisés
J989	Trouble respiratoire, sans précision
J998	Troubles respiratoires au cours d’autres maladies classées ailleurs
K290	Gastrite hémorragique aiguë
K291	Autres gastrites aiguës
K296	Autres gastrites
K297	Gastrite, sans précision
K298	Duodénite
K299	Gastroduodénite, sans précision
K523	Colite indéterminée
K528	Autres gastroentérites et colites non infectieuses précisées
K529	Gastroentérite et colite non infectieuses, sans précision
K591	Diarrhée fonctionnelle
P220	Syndrome de détresse respiratoire du nouveau-né (SDR)
P221	Tachypnée transitoire du nouveau-né
P228	Autres détresses respiratoires du nouveau-né
P229	Détresse respiratoire du nouveau-né, sans précision
P230	Pneumonie congénitale due à un agent viral
P231	Pneumonie congénitale à Chlamydia
P232	Pneumonie congénitale à staphylocoques
P233	Pneumonie congénitale due à des streptocoques, groupe B
P234	Pneumonie congénitale à *Escherichia coli*
P235	Pneumonie congénitale à Pseudomonas
P236	Pneumonie congénitale due à d’autres agents bactériens
P238	Pneumonie congénitale due à d’autres micro-organismes
P239	Pneumonie congénitale, sans précision
P280	Atélectasie primitive du nouveau-né
P281	Atélectasies du nouveau-né, autres et sans précision
P282	Crises de cyanose du nouveau-né
P283	Apnée primitive du sommeil chez le nouveau-né
P284	Autres apnées du nouveau-né
P285	Insuffisance respiratoire du nouveau-né
P288	Autres affections respiratoires précisées chez le nouveau-né
P289	Affection respiratoire du nouveau-né, sans précision
P2918	Autre dysrythmie cardiaque néonatale
P358	Autres maladies virales congénitales
P359	Maladie virale congénitale, sans précision
P368	Autre sepsie bactérienne du nouveau-né
P369	Sepsie bactérienne du nouveau-né, sans précision
P741	Déshydratation du nouveau-né
R000	Tachycardie, sans précision
R002	Palpitations
R008	Anomalies des battements cardiaques, autres et non précisées
R030	Constatation d’une élévation de la tension artérielle, sans diagnostic d’hypertension
R031	Constatation d’une baisse non spécifique de la tension artérielle
R05	Toux
R060	Dyspnée
R061	Stridor
R062	Sifflement
R063	Respiration périodique
R064	Hyperventilation
R065	Respiration par la bouche
R067	Éternuement
R068	Anomalies de la respiration, autres et non précisées
R070	Douleur de la gorge
R071	Douleur thoracique respiratoire
R072	Douleur précordiale
R073	Autres douleurs thoraciques
R074	Douleur thoracique, sans précision
R093	Expectoration anormale
R098	Autres symptômes et signes précisés relatifs aux appareils circulatoire et respiratoire
R100	Syndrome abdominal aigu
R1010	Douleur localisée au quadrant supérieur droit
R1011	Douleur localisée au quadrant supérieur gauche
R1012	Douleur épigastrique
R1019	Douleur localisée à la partie supérieure de l’abdomen, sans précision
R1030	Douleur localisée au quadrant inférieur droit
R1031	Douleur localisée au quadrant inférieur gauche
R1032	Douleur périombilicale
R1039	Douleur localisée à la partie inférieure de l’abdomen, sans précision
R104	Douleurs abdominales, autres et non précisées
R110	Vomissement en jet
R111	Nausées seules
R112	Vomissements seuls
R113	Nausées avec vomissements
R130	Dysphagie oro-pharyngée
R132	Dysphagie oesophagienne
R138	Dysphagie, autre et non précisée
R508	Autre fièvre précisée
R509	Fièvre, sans précision
R51	Céphalée
R520	Douleur aiguë
R529	Douleur, sans précision
R53	Malaise et fatigue
R5601	Convulsions fébriles complexes
R5602	Convulsions fébriles simples
R5609	Convulsions fébriles, sans précision
R5680	Trouble convulsif, décrit ainsi
R5688	Convulsions, autres et non précisées
R571	Choc hypovolémique
R572	Choc septique
R578	Autre choc
R579	Choc, sans précision
R590	Adénopathies localisées
R591	Adénopathies généralisées
R599	Adénopathie, sans précision
R650	Syndrome de réponse inflammatoire systémique d’origine infectieuse sans défaillance organique
R651	Syndrome de réponse inflammatoire systémique d’origine infectieuse avec défaillance organique aiguë
R652	Syndrome de réponse inflammatoire systémique d’origine non infectieuse sans défaillance organique
R653	Syndrome de réponse inflammatoire systémique d’origine non infectieuse avec défaillance organique aiguë
R659	Syndrome de réponse inflammatoire systémique, non spécifié
R91	Résultats anormaux d’imagerie diagnostique du poumon
U0490	Syndrome respiratoire aigu sévère [SRAS] suspect
U0491	Syndrome respiratoire aigu sévère [SRAS] probable
U071	COVID-19 virus identifié
U071NV	COVID-19 virus identifié avec ventilation
U071S	COVID-19 virus identifié avec admission aux soins intensifs
U071SV	COVID-19 virus identifié avec admission aux soins intensifs et ventilation
U072	COVID-19, virus non identifié
U072NV	COVID-19 virus non identifié avec ventilation
U072S	COVID-19 virus non identifié avec admission aux soins intensifs
U072SV	COVID-19 virus non identifié admission aux soins intensifs et ventilation
U073	Syndrome inflammatoire multisystémique associé à la COVID-19
U074	Affection post-COVID-19
Z038	Mise en observation pour suspicion d'autres maladies et affections
Z039	Mise en observation pour suspicion de maladie ou affection, sans précision
Z048	Examen et mise en observation pour d'autres raisons précisées
Z049	Examen et mise en observation pour une raison non précisée
Z519	Soin médical, sans précision

**Table A2:** Number of intensive care unit admissions and in-hospital deaths by age group, sex, vaccination status, prior infection and presence of comorbidities

Variable	January 2 to 29, 2022					January 30 to February 26, 2022					February 27 to March 26, 2022					March 27 to April 23, 2022				
	Hospital admissions	ICU		In-hospital deaths		Hospital admissions	ICU		In-hospital deaths		Hospital admissions	ICU		In-hospital deaths		Hospital admissions	ICU		In-hospital deaths	
		N	% (95% CI)	N	% (95% CI)		N	% (95% CI)	N	% (95% CI)		N	% (95% CI)	N	% (95% CI)		N	% (95% CI)	N	% (95% CI)
Global	4,216	565	13.4 [12.4–14.4]	844	20 [18.8–21.2]	1,550	187	12.1 [10.4–13.7]	241	15.5 [13.7–17.4]	1,015	89	8.8 [7–10.5]	150	14.8 [12.6–17]	2,397	185	7.7 [6.6–8.8]	288	12 [10.7–13.3]
**Age group (years)**																				
0–45	469	65	13.9 [10.7–17]	5	1.1 [0.1–2]	247	24	9.7 [6–13.4]	3	1.2 [0–2.6]	121	7	5.8 [1.6–9.9]	1	0.8 [0–2.4]	248	24	9.7 [6–13.4]	5	2 [0.3–3.8]
44–55	254	50	19.7 [14.8–24.6]	11	4.3 [1.8–6.8]	89	18	20.2 [11.9–28.6]	5	5.6 [0.8–10.4]	39	8	20.5 [7.8–33.2]	2	5.1 [0–12.1]	68	4	5.9 [0.3–11.5]	0	0 [0–0]
56–65	544	131	24.1 [20.5–27.7]	60	11 [8.4–13.7]	171	34	19.9 [13.9–25.9]	16	9.4 [5–13.7]	103	18	17.5 [10.1–24.8]	10	9.7 [4–15.4]	200	28	14 [9.2–18.8]	15	7.5 [3.8–11.2]
66–75	911	176	19.3 [16.8–21.9]	161	17.7 [15.2–20.1]	316	57	18 [13.8–22.3]	51	16.1 [12.1–20.2]	186	27	14.5 [9.5–19.6]	28	15.1 [9.9–20.2]	446	60	13.5 [10.3–16.6]	43	9.6 [6.9–12.4]
Over 75	2,038	143	7 [5.9–8.1]	607	29.8 [27.8–31.8]	727	54	7.4 [5.5–9.3]	166	22.8 [19.8–25.9]	566	29	5.1 [3.3–6.9]	109	19.3 [16–22.5]	1,435	69	4.8 [3.7–5.9]	225	15.7 [13.8–17.6]
**Sex**																				
Male	2,252	350	15.5 [14–17]	502	22.3 [20.6–24]	809	108	13.3 [11–15.7]	130	16.1 [13.5–18.6]	538	55	10.2 [7.7–12.8]	88	16.4 [13.2–19.5]	1,243	103	8.3 [6.8–9.8]	166	13.4 [11.5–15.2]
Female	1,964	215	10.9 [9.6–12.3]	342	17.4 [15.7–19.1]	741	79	10.7 [8.4–12.9]	111	15 [12.4–17.5]	477	34	7.1 [4.8–9.4]	62	13 [10–16]	1,154	82	7.1 [5.6–8.6]	122	10.6 [8.8–12.3]
**Vaccination**																				
Adequate	2,864	312	10.9 [9.8–12]	628	21.9 [20.4–23.4]	1,027	96	9.3 [7.6–11.1]	171	16.7 [14.4–18.9]	785	63	8 [6.1–9.9]	126	16.1 [13.5–18.6]	1940	137	7.1 [5.9–8.2]	246	12.7 [11.2–14.2]
Inadequate	1,348	251	18.6 [16.5–20.7]	216	16 [14.1–18]	519	89	17.1 [13.9–20.4]	69	13.3 [10.4–16.2]	229	26	11.4 [7.2–15.5]	24	10.5 [6.5–14.4]	453	47	10.4 [7.6–13.2]	42	9.3 [6.6–11.9]
Missing	4	2	50 [1–99]	0	0 [0–0]	4	2	50 [1–99]	1	25 [0–67.4]	1	0	0 [0–0]	0	0 [0–0]	4	1	25 [0–67.4]	0	0 [0–0]
**Prior infection according to laboratory tests**																				
No	4,151	563	13.6 [12.5–14.6]	837	20.2 [18.9–21.4]	1,520	182	12 [10.3–13.6]	239	15.7 [13.9–17.6]	993	89	9 [7.2–10.7]	147	14.8 [12.6–17]	2,338	185	7.9 [6.8–9]	284	12.1 [10.8–13.5]
Yes	65	2	3.1 [0–7.3]	7	10.8 [3.2–18.3]	30	5	16.7 [3.3–30]	2	6.7 [0–15.6]	22	0	0 [0–0]	3	13.6 [0–28]	59	0	0 [0–0]	4	6.8 [0.4–13.2]
**Comorbidities**																				
None	521	104	20 [16.5–23.4]	38	7.3 [5.1–9.5]	219	29	13.2 [8.8–17.7]	13	5.9 [2.8–9.1]	122	10	8.2 [3.3–13.1]	7	5.7 [1.6–9.9]	236	30	12.7 [8.5–17]	18	7.6 [4.2–11]
At least one	3,526	448	12,7 [11,6–13,8]	798	22,6 [21,3–24]	1,247	152	12.2 [10.4–14]	225	18 [15.9–20.2]	846	77	9.1 [7.2–11]	140	16.5 [14–19.1]	2,066	148	7.2 [6.1–8.3]	267	12.9 [11.5–14.4]
Missing	169	13	7,7 [3,7–11,7]	8	4,7 [1,5–7,9]	84	6	7.1 [1.6–12.7]	3	3.6 [0–7.5]	47	2	4.3 [0–10]	3	6.4 [0–13.4]	95	7	7.4 [2.1–12.6]	3	3.2 [0–6.7]

Abbreviations: CI, confidence interval; ICU, intensive care unit

**Table A3:** Percentiles for the length of stay in hospital (days), by age group, sex, vaccination status, prior infection and presence of comorbidities

Variable	January 2 to 29, 2022				January 30 to February 26, 2022				February 27 to March 26, 2022				March 27 to April 23, 2022			
	Hospital admissions	Percentile			Hospital admissions	Percentile			Hospital admissions	Percentile			Hospital admissions	Percentile		
		25^th^	50^th^	75^th^		25^th^	50^th^	75^th^		25^th^	50^th^	75^th^		25^th^	50^th^	75^th^
Global	4,216	3	7	15	1,550	3	6	14	1,015	3	7	16.5	2,397	3	7	14
**Age group (years)**																
0–45	469	1	2	6	247	1	2	4	121	1	2	4	248	1	2	3
44–55	254	3	5	9	89	2	4	8	39	3	6	13.5	68	2	4	10.5
56–65	544	4	7	15	171	3	7	14	103	3.5	7	17	200	2	4.5	12.3
66–75	911	4	8	17	316	3	7	15	186	4	9	17.75	446	3	6	12
Over 75	2,038	4	8	17	727	4	9	18	566	4	9	19	1,435	4	9	17
**Sex**																
Male	2,252	3	7	15	809	2	6	13	538	3	7	15	1,243	3	7	14.5
Female	1,964	3	7	14	741	3	7	15	477	3	7	17	1,154	3	6	13
**Vaccination**																
Adequate	2,864	4	7	15	1,027	3	7	15.5	785	4	8	18	1,940	3	7	14
Inadequate	1,348	3	6	13	519	2	5	11	229	2	4	11	453	2	5	14
Missing	4	–	–	–	4	–	–	–	1	–	–	–	4	–	–	–
**Prior infection according to laboratory tests**																
No	4,151	3	7	15	1,520	3	6	14	993	3	7	16	2,338	3	7	14
Yes	65	4	8	11	30	4	8	11	22	2	6	21.75	59	3	7	15
**Comorbidities**																
None	521	2	5	11	219	1	3	10	122	2	4	10	236	1	3	10
At least one	3,526	4	7	16	1,247	3	7	15.5	846	4	8	19	2,066	3	7	15
Missing	169	1	2	4	84	1	2	3	47	1	2	4.5	95	1	2	3
